# Method of calculating shear strength of rock mass joint surface considering cyclic shear degradation

**DOI:** 10.1038/s41598-022-13505-6

**Published:** 2022-06-07

**Authors:** Shan Dong, Heng Zhang, Yulin Peng, Zhichun Lu, Weihua Hou

**Affiliations:** 1 Badong National Observation and Research Station of Geohazards, China University of Geosciences, Wuhan, 430074 China; 2 Three Gorges Research Center for Geohazards, China University of Geosciences, Wuhan, 430074 China

**Keywords:** Civil engineering, Solid Earth sciences

## Abstract

When a rock mass shears along a joint surface, the shear resistance is affected by joint surface undulations and friction between the contact regions. During an earthquake, the seismic load causes dynamic deterioration of the joint surface mechanical properties, mostly reflected as follows. (1) The peak shear strength of the joint surface decreases with an increase in the shear rate. (2) Under a seismic load cyclic shear, the undulant angle *α*_*k*_ decreases. (3) Under a dynamic load, the friction coefficient of the joint surface is reduced. By studying the cyclic shear test of the joint surface, the strength deterioration effect of the joint surface under cyclic shearing is first analysed, and the equations of the dilatation angle and the basic friction angle of the joint surface under the cyclic shearing load are proposed. Then, starting with the effect of cyclic shear deterioration on the joint surface in the rock mass and the reduction in the dynamic friction coefficient between sliding rock blocks caused by relative velocity, an equation for calculating the shear strength of a rock mass joint surface under cyclic shear loading is recommended. Through two case calculations, the shear strength obtained using the proposed method is compared with the experimental results. The results show that the model proposed in this study is in good agreement with the experimental results and can also be used to calculate the structural surface shear strength of the asperity-rich sample. However, when the calculation equation is used to estimate the cyclic shear strength of the joint surface where the sum of the initial undulation angle and the basic friction angle is greater than 70°, there may be some errors in the calculation results.

## Introduction

As a type of dynamic load, the magnitude and direction of earthquake acceleration vary constantly. For joint surfaces, seismic loads are both dynamic and cyclic. According to previous studies^1–4^, when a rock mass shears along a joint surface, the shear resistance of the joint surface is affected by undulations and friction between the contact regions. Several rock mass dynamic tests have indicated that, under cyclic loading, the joint surface gradually degrades^5–11^. The influence of the seismic load on the joint surface strength properties manifests in two ways: (1) The relative velocity effect and seismic vibration wear effect. Under seismic loading, the relative velocity generated by the rock mass movement reduces the frictional coefficient of the joint surface, and a functional relationship exists between the dynamic frictional coefficient and the relative motion velocity. (2) Under a seismic cyclic shear load, the joint surface undulations are worn and sheared, and the sheared undulations are filled inside the joint surface as debris. Therefore, the shear of the joint surface under a seismic cyclic load directly changes the friction angle of the rock mass joint surface. The degradation effect of the joint surface frictional angle must be considered when calculating the joint surface shear strength under a cyclic seismic shear load^12–16^. Joint surface degradation may lead to slope rock mass movement along the joint surface, eventually causing instability and resulting in bedding rock landslides. Therefore, studying the degradation mechanism of the rock mass joint surface shear strength under cyclic loading is a key scientific issue that must be resolved to accurately evaluate the dynamic stability of slope rock masses.

Wang and Zhang^1^ studied the movement characteristics of a non-undulating granite joint surface under low-frequency vibrations and the changes in the frictional characteristics of the sliding surface during block motion. They suggested that the initial frictional force is related to the loading waveform curve from static to dynamic loading; that is, the initial frictional force is affected by the loading rate or vibration frequency. After the block starts moving, the dynamic frictional coefficient is related to the relative movement velocity and its accumulated displacement. Crawford^17,18^ performed a dynamic test on a rock mass and revealed that the seismic yield acceleration of a flat joint surface varied with the cumulative displacement and velocity of rock mass movement. Jafari et al.^5^ conducted shear tests on artificial sawtooth rock mass joint surface samples at different shear rates and inferred that the peak shear strength of the joint surface decreased with increasing shear rate. Through a shear test of the rock mass joint surface, Li et al.^19^ inferred that the peak shear strength decreases with an increase in shear deformation velocity, and that the decreasing rates decrease with increasing shearing deformation velocity. Atapour et al.^9^ conducted shear tests on concrete joint surfaces at different shear rates and concluded that the friction angle increased with increasing shear rate.

The accurate evaluation of the shear strength of rough rock joints has always been pursued by rock mechanics workers^20^. Several scholars have proposed various shear constitutive models for cyclic and dynamic load conditions based on test results and elastoplastic theory. For example, Hutson and Dowding^21^ performed some cyclic tests on artificial sinusoidal joints and proposed a wear equation for joint roughness based on the test results. Huang et al.^22^ performed cycle tests on sawtooth samples and verified the degradation law proposed by Plesha^23^. Souley et al.^24^ approximated the tangential stress–strain relationship with a piecewise linear function. Qiu et al.^25^ regarded structural surface wear as plastic deformation and proposed a tangential plastic work function as an index to express wear and established an elastoplastic constitutive model considering the dilatancy angle and wear parameters. Kana et al.^26^ proposed an interlocking friction model through joint surface cyclic shear testing. Divoux et al.^27^ proposed a cyclic shear mechanics constitutive model for joint surfaces based on the results of numerous cyclic shear tests. Mroz et al.^28^ described microscopic effects such as debonding, slippage, and dilatancy of the micro convex body through the interaction of spherical structural surfaces and proposed a constitutive model that can effectively simulate the softening phenomenon after the first cycle. Evidently, although numerous rock mass dynamic tests have indicated that under cyclic loading, rock mass joint surface degradation is complex, several significant results pertaining to the study of the cyclic load shear strength of the joint surface have been achieved. However, there are few quantitative and systematic research results on the analysis of the rock shear constitutive model joint surface considering both the shear rate effect and shear friction angle degradation effect.

In this study, the deterioration effect of the joint surface shear strength under cyclic shearing is first analysed through a cyclic shear test of the joint surface, and the dilatation angle equation of the joint surface under a cyclic shearing load is proposed. On this basis, a calculation method for the basic friction angle of cyclic shearing of the joint surface is proposed. Furthermore, a shear strength calculation equation is proposed, considering the effect of vibration deterioration on the joint surface and the reduction in the dynamic friction coefficient between sliding rock blocks caused by relative velocity.

## Deterioration of the joint surface under cyclic loading

### Experimental method

The joint surface morphology is the main factor controlling its mechanical properties. Natural joint surfaces with complex surface undulations are difficult to sample and the sample consistency cannot be guaranteed. Preparing rock mass joint surface samples with similar materials has proven to be feasible, especially the artificial sawtooth joint surface can quantitatively describe the effect of the undulant angle on the joint surface mechanical properties^29–32^. At present, the shear characteristics of joint surfaces with different undulations are mainly studied using artificial sawtooth joint surfaces^33,34^.

**Figure 1:**
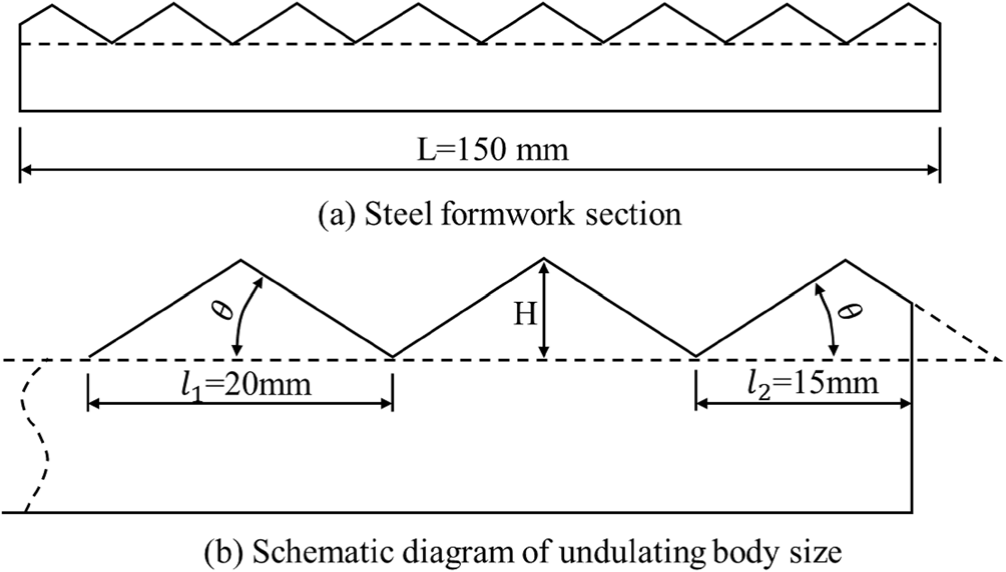
Amplitude of artificial joint surface.

Liu et al.^35,36^ prepared rock mass joint surface samples with first-order undulation angles of 16°, 25°, 33°, 40°, and 45° and joint wall strengths of 10, 20, and 30 MPa, using cement mortar as a similar material. According to the experimental design, five steel moulds with different undulating angles and pouring moulds with a specific height were pre-processed. The upper and lower parts of the joint surface sample were prepared by the pouring method, and the two parts were combined to form a complete joint sample. The total length of the joint surface sample was 150 mm, the sawtooth protrusion adopted a fixed step length (20 mm), and the difference in undulation was reflected by the height (H) change in the sawtooth protrusions (Fig. 1).

The mortar aggregate was fine sand, which was fully stirred and vibrated during the pouring process to ensure that the strength of the joint surface protrusion and the strength of the block were consistent. After the sample was poured and cured, a uniaxial test was carried out using the inspection samples poured in the same batch to check the compressive strength value of the joint sample material. Table 1 lists the compressive strength test results of the joint surface sample materials. The test results show that the strengths of the joint surface samples of the same strength grade have good consistency, and the error range is within 7%, which meets the test requirements.

**Table 1:** Compressive strength test results of joint surface materials.

No	Measured value of compressive strength/MPa		
	10 MPa	20 MPa	30 MPa
1	10.30	21.00	32.00
2	9.80	20.40	31.80
3	10.40	20.50	29.20
4	9.70	19.70	29.60
5	9.80	19.50	30.40
6	10.20	20.60	30.40
7	9.90	19.20	29.80
8	9.60	19.80	30.50
Average value	9.96	20.09	30.46

### Test device and single cycle routes

**Figure 2:**
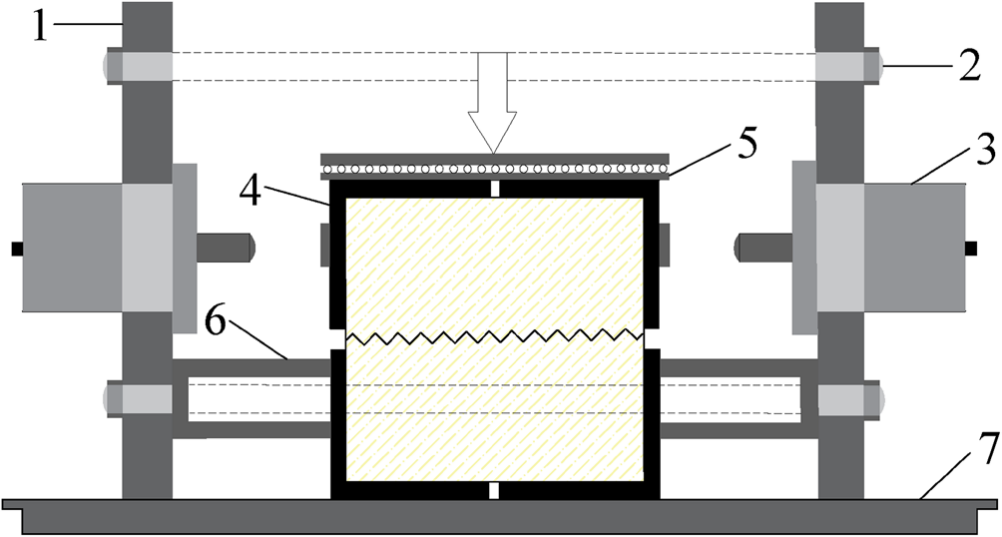
RJDT-A shear box schematic diagram. 1—Frame baffle; 2—Fixed screw; 3—Jack loading; 4—Shear box; 5—Skateboards; 6—Fixed roof; 7—Bottom board.

**Figure 3:**
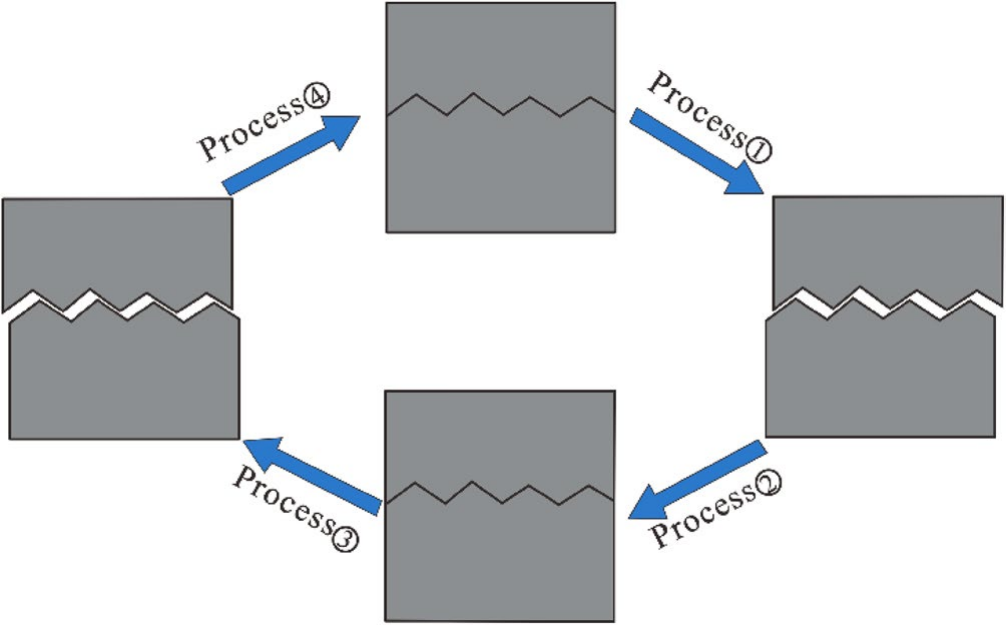
Cyclic shear process of the joint surface.

Cyclic shear tests on joint surfaces with normal stresses of 0.8 MPa, 1.6 MPa, 2.4 MPa, and 3.2 MPa were carried out using the RJDT-A shear testing machine independently developed by the Institute of Rock and Soil Mechanics, Chinese Academy of Sciences (Fig. 2). During the test, the shearing force was applied by the jacks at both ends of the frame, the shearing direction was controlled by the oil circuit, and normal servo jacks applied to the normal stress of the joint specimen. The maximum shear displacement in the positive and negative test directions was taken as 1/2 of the protrusion step length (10 mm). The cyclic shearing process of the joint specimens is shown in Fig. 3. During the test, the lower shear box was fixed and the route division was based on the shear displacement of the upper shear box. Initially, the upper and lower parts of the joint surface sample were stationary. Next, the upper part was sheared to the right to + 10 mm (process ①); then, the shearing direction was changed, and sheared to the left until the displacement was − 10 mm (process ②, ③); then, the shearing direction was changed to the right and cut to a displacement of 0 mm (process ④), and a cyclic shearing process was completed (Fig. 3).

### Shear strength deterioration effect

Liu et al.^35,36^ demonstrated the deterioration law of joint surface shear strength by defining the shear strength deterioration index of the joint surface shear strength ratio. The joint surface shear strength ratio (*D*_*n*_, with a value between 0.0 and 1.0) refers to the ratio of the peak shear strength of the *n-*th shear cycle (${\tau }_{n}$) to the peak shear strength of the first shear cycle (${\tau }_{1}$).

1 $${D}_{n}=\frac{{\tau }_{n}}{{\tau }_{1}}.$$

The closer ${D}_{n}$ is to 1, the smaller the strength deterioration caused by the cyclic shear of the joint surface. The smaller the ${D}_{n}$, the greater the strength deterioration caused by the cyclic shear of the joint surface.

**Figure 4:**
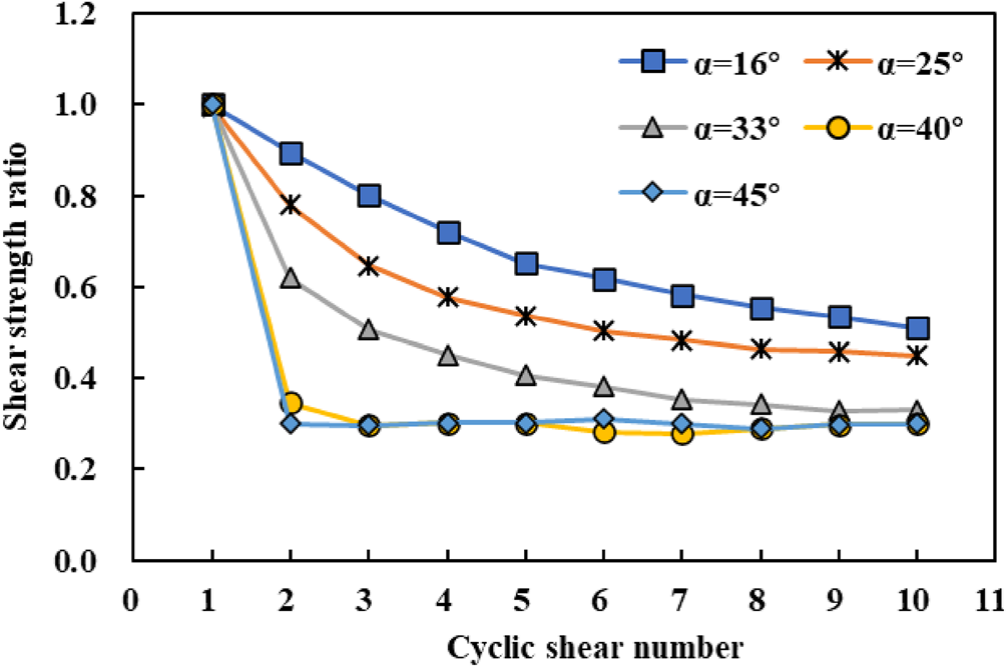
Change in shear strength ratio of joint surfaces with different undulation angles with cyclic shear times under a normal stress of 0.8 MPa.

The experimental results show that the variation in joint surface shear strength ratio (${D}_{n}$) is essentially the same under the action of different normal stresses^35,36^. This study takes the test results of the normal stress of 0.8 MPa as an example to analyse the shear strength deterioration of the joint surface during the cyclic shearing process. Figure 4 shows the changes in the shear strength ratios of joint surfaces with different undulation angles with cyclic shear times under a normal stress of 0.8 MPa. The experimental results show that the peak shear strengths of the joint surface samples with different undulation angles decreased with increasing cyclic shear times, although the decreasing trend differed. For example, the shear strength ratio of joint surface samples with an undulation angle of 16° decreased at the lowest rate with increasing cyclic shear times, whereas the shear strength of a joint surface with an undulation angle of 45° decreased at the highest rate with increasing cyclic shear times. In general, the larger the undulation angle, the faster the shear strength ratio decreases with an increase in the cyclic shear times. This is because the higher the protrusions on the joint surface, the lower their shear resistance, and large-area shear damage during the re-shearing process is more likely. Therefore, for a joint surface with higher undulation angles, damage to the surface protrusions mainly occurs in the first or initial shear cycles, and the degree of damage in the subsequent shear cycles does not significantly change. For a joint surface with smaller undulation angles, protrusion damage is mainly caused by continuous wear or multiple shear failures, requiring cumulative failure through multiple shear cycles to achieve residual strength. Therefore, the shear-strength ratio of the joint surface with smaller undulation angles decreased more slowly as the number of cycles increased.

### Equation for calculating the shear strength

Newland and Allely^37^ developed the following equation for the shear strength of a joint surface:

2 $$\tau ={\sigma }_{n}\text{tan}\left({\varphi }_{b}+i\right).$$

Partton^38,39^ and Goldstein et al.^40^ used Eq. (2) to represent the shear strength of irregular rock mass joint surfaces and broken rock when tested at low normal stresses. At high normal stresses, it is assumed that the Coulomb relationship is:

3 $$\tau =c+{\sigma }_{n}\text{tan}\varphi .$$

In Eqs. (2) and (3), τ is the shear stress, ${\sigma }_{n}$ is the effective normal stress, *i* is the average deviation angle of particle displacements from the applied shear stress direction, ${\varphi }_{b}$ is the basic friction angle, c is the cohesion, ${\sigma }_{n}$ is the normal stress, and φ is the total friction angle.

In fact, the peak shear strength envelopes for non-planar rock joints are nonlinear, and there are some differences between the Coulomb relationship and the actual situation of rough joint surface shearing^41–43^. An empirical non-linear equation of peak shear strength that is sensitive both to variable joint roughness and to variable compressive strength for the rock or joint walls is proposed by Barton^41^ through a large number of joint shear tests, and the empirical non-linear equation is as follows:

4 $$\uptau ={\sigma }_{n}\text{tg}\left(JRC\cdot \text{lg}\frac{JCS}{{\sigma }_{n}}+{\varphi }_{b}\right).$$

The peak shear strength of a rock joint, as described in Eq. (4) can also be expressed as follows:

5 $$\uptau ={\sigma }_{n}\text{tan}\left({\alpha }_{p}+{S}_{a}+{\varphi }_{b}\right).$$

In Eqs. (4) and (5), where *JRC* denotes the joint roughness coefficient, *JCS* denotes the joint wall compressive strength, ${\varphi }_{b}$ denotes the basic friction angle, ${\alpha }_{p}$ denotes the peak dilatancy angle, and ${S}_{a}$ denotes the asperity failure component. Because the ${S}_{a}$ is of a similar magnitude to the dilation angle^42,44^, Eq. (5) can be expressed as follows:

6 $$\uptau ={\sigma }_{n}\text{tan}\left(2{\alpha }_{p}+{\varphi }_{b}\right).$$

Therefore, the cyclic shear strength of joint surface can be expressed as

7 $${\uptau }_{n}={\sigma }_{n}\text{tan}\left(2{\alpha }_{n}+{\varphi }_{n}\right),$$

where ${\alpha }_{n}$ is the dilatancy angle of the nth cyclic shear and ${\varphi }_{n}$ is the basic friction angle of the joint surface of the nth cyclic shear. According to Eq. (7), when the normal stress and shear rate are constant, with an increase in the number of cycles, the shear stress is mainly affected by the dilatancy and basic friction angles. Therefore, clarifying the changes in the dilatancy angle and basic friction angle in the cyclic shear process is the key to solving the shear strength of the joint surface in the cyclic shear process.

## Dilatancy angle and basic frictional angle

### Dilatancy angle of the joint surface

**Figure 5:**
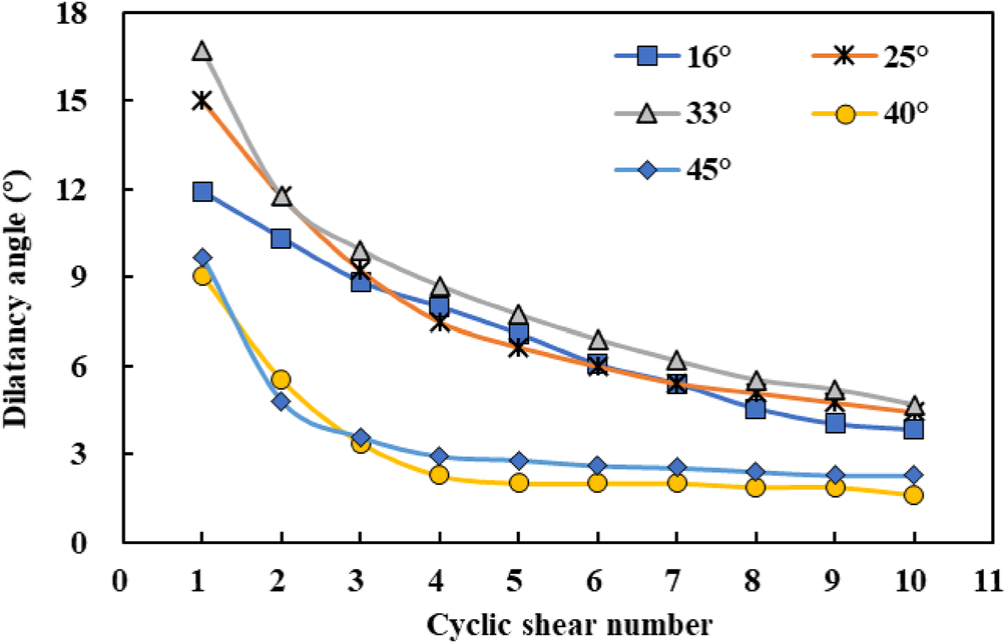
Dilatancy angle changes with shear cycles under different initial undulation angles.

Figure 5 shows the dilatancy angle changes with shear cycles under different initial undulation angles. With an increase in the initial undulation angle, the dilatation angle of the first shear cycle of the joint surface initially increased and then decreased (45° < 40° < 16° < 25° < 33°). This is because the shear failure modes differ between joint surfaces with high and medium undulation angles. Under the same normal stress, as the undulant angle of the joint surface increased, the sliding failure of the rock mass during the shearing process gradually weakened, the shear failure gradually increased, and the degree of joint surface deterioration intensified. When the undulation angle reached 40° and 45°, the phenomenon of compressive tensile failure was gradually obvious. This means that the larger undulant angle of the joint surface, the more severe the damage during the shearing process, and the undulation angle of the 40° and 45° joint surfaces is more extreme. However, in general, for the joint surfaces with different initial undulation angles, with an increase in the number of cyclic shears, the dilation angle first decreases sharply, then decreases gradually, and tends to stabilise slowly.

Based on these tests, Liu et al.^36^ found that the dilatancy angle of a joint surface under cyclic shear conforms to a negative exponential distribution and proposed the following expression:

8 $${\alpha }_{n}={\alpha }_{0}\left(A{e}^{-n/B}+C\right),$$

where ${\alpha }_{n}$ denotes the dilatancy angle of the n-th cyclic shear, ${\alpha }_{0}$ denotes the initial undulation angle of the joint surface, and *n* denotes the number of shear cycles. Notably, when the normal stress is zero or the joint surface is not subjected to shear, ${\alpha }_{n}$ = ${\alpha }_{0}$. Thus, the initial value of ${\alpha }_{n}$ was ${\alpha }_{0}$.

The wear coefficient of the dilatancy angle is further defined as follows:

9 $${R}_{s}=\frac{{\alpha }_{n}}{{\alpha }_{0}}=A{e}^{-n/B}+C.$$

When $n=0$, ${R}_{s}=1$, and $A+C=1$, and Eq. (9) can be expressed as follows:

10 $${R}_{s}=A{e}^{-n/B}+C=A\left({e}^{-n/B}-1\right)+1.$$

Thus,

11 $${\alpha }_{n}={\alpha }_{0}{R}_{s}={\alpha }_{0}\left[A\left({e}^{-n/B}-1\right)+1\right].$$

The values of *A* and *B* of the joint surface samples with the same intact rock strength have a linear relationship with the normal stress; therefore, it is assumed that the calculation formulas of parameters *A* and *B* are:

12 $$A=a{\sigma }_{n}+b,$$

13 $$B=c{\sigma }_{n}+d,$$

where *a*, *b*, *c*, and *d* are constants related to intact rock strength ${\sigma }_{c}$. The relationships among *a*, *b*, *c*, and *d* and the intact rock strength ${\sigma }_{c}$ were therefore regressed.

### Basic frictional angle of the joint surface

Based on the generalised failure model, Dong et al.^45^ proposed that if the shear dilatancy angle (${\alpha }_{n}$) is similar to the initial undulant angle (${\alpha }_{0}$), the joint surface is subjected to less shear failure, and the basic frictional angle (${\varphi }_{n}$) is similar to the initial basic frictional angle (${\varphi }_{0}$). Similarly, if the shear dilatancy angle (${\alpha }_{n}$) is closer to the residual undulant angle (${\alpha }_{r}$), the joint surface is severely damaged by cyclic shear, and the basic frictional angle (${\varphi }_{n}$) is also similar to the residual basic frictional angle (${\varphi }_{r}$). It was inferred that the basic friction angle $\left({\varphi }_{n}\right)$ changes in the interval of [${\varphi }_{0}, {\varphi }_{r}$] during cyclic shear is similar to that of the dilatancy angle ${\alpha }_{n}$ in the interval of [${\alpha }_{0}, {\alpha }_{r}$]. Therefore, the following equation is obtained:

14 $$\frac{{\alpha }_{0}-{\alpha }_{n}}{{\alpha }_{0}-{\alpha }_{r}}=\frac{{\varphi }_{0}-{\varphi }_{n}}{{\varphi }_{0}-{\varphi }_{r}},$$

where ${{\alpha }}_{\text{n}}$ denotes the dilatancy angle of the joint surface of the n-th cyclic shear, ${\alpha }_{0}$ denotes the initial dilatancy angle, ${\alpha }_{r}$ denotes the residual dilatancy angle, ${\varphi }_{0}$ denotes the initial basic friction angle of the joint surface, ${\varphi }_{r}$ denotes the residual basic friction angle, and ${\varphi }_{n}$ denotes the basic friction angle of the joint surface of the n-th cyclic shear.

Based on Eq. (14), the basic joint surface frictional angle after the nth cyclic shear (${\varphi }_{n}$) can be defined as follows:

15 $${\varphi }_{n}=\frac{{\alpha }_{n}\left({\varphi }_{0}-{\varphi }_{r}\right)-{\varphi }_{0}{\alpha }_{r}+{\varphi }_{r}{\alpha }_{0}}{{\alpha }_{0}-{\alpha }_{r}},$$

where ${{\varphi }}_{0}$can be determined based on the inclination angle test using a smooth test block.

Based on Eq. (7), the residual basic frictional angle (${{\varphi }}_{\text{r}}$) can be expressed as

16 $${\varphi }_{r}=\text{arctan}\left({\tau }_{r}/{\sigma }_{n}\right)-{2\alpha }_{r},$$

where ${\uptau }_{\text{r}}$ denotes the residual shear stress. Then, based on Eqs. (15) and (16), the basic frictional angle can be determined as follows:

17 $${\varphi }_{n}=\frac{\left({\alpha }_{0}-{\alpha }_{n}\right)\left[\text{arctan}\left({\tau }_{r}/{\sigma }_{n}\right)-{2\alpha }_{r}\right]+\left({\alpha }_{n}-{\alpha }_{r}\right){\varphi }_{0}}{{\alpha }_{0}-{\alpha }_{r}}.$$

## Relative velocity effect

Based on the peak shear strength theory proposed by Barton^41^ and the joint roughness coefficient calculation method of the root mean square first-order derivative method, Zheng et al.^46^ sorted and calculated many direct shear test results of rock joints with different shear rates under constant normal stress and explored the influence of shear rate on the total friction angle of rock joints. It was found the shear rate has a greater impact on the total friction angle when the shear rate is in the range of 0–0.8 mm s^−1^, and the shear rate has a greater impact on the total friction angle. For joints with strong homogeneity and isotropy, the total friction angle decreases with an increase in shear rate, such as gypsum, cement, and sandstone joints. For joints with strong heterogeneity and anisotropy, the total friction angle increases with the increase in shear rate, such as concrete and syenite joints, and the increase in the total friction angle of the latter is smaller than the decrease in the former. In this study, homogeneous and isotropic joints are considered, and the case in which the total friction angle decreases with the increase in shear rate is considered.

Through dynamic rock block tests, Wang and Zhang^1^ observed that the frictional coefficient of the structural plane was not constant throughout the block movement and varied dynamically with the relative movement rate and cumulative displacement. Evidently, after the block started moving, the coefficient of dynamic friction was related to the relative movement speed and cumulative displacement. The dynamic friction coefficient (${f}_{d}$) of the structural plane is expressed as

18 $${f}_{d}={f}_{s}\cdot {f}_{c}\left(\dot{x}\right),$$

where ${f}_{c}(\dot{x})$ is a function that decreases with an increase in $\left|\dot{x}\right|$. When *ẋ* = 0, ${f}_{c}\left(\dot{x}\right)=1$. $\dot{x}$ is the velocity of the upper block relative to the base, and ${f}_{s}$ is known as the starting frictional coefficient that is related to the cumulative displacement. Because the cumulative displacement affects the smoothness of the rock mass contact plane, ${f}_{s}$ is a decreasing function with increasing cumulative displacement.

Li et al.^19^ experimentally studied the influence of shear rate on the shear strength of rock mass joint surfaces and inferred that the peak shear strength decreases with increasing shear rate and the relationship is described by an exponential decay function. Based on the studies of Wang and Zhang^1^ and Li et al.^19^, Liu^47^ proposed an expression for the relative velocity factor, which can be expressed as follows:

19 $$\upgamma \left(t\right)={\gamma }_{r}+\left(1-{\gamma }_{r}\right){e}^{-a\left|\dot{x}\left(t\right)\right|},$$

where $\dot{x}\left(t\right)$ is the relative velocity between the upper and lower rock blocks on the joint surface, ${\gamma }_{r}$ is is the convergence value of the relative velocity influence factor, and a is an undetermined coefficient. Based on the results obtained by Li, ${\gamma }_{r}=0.9\, \text{and}\, a = 25$.

Based on Eqs. (7), (11), and (17), the shear strength of the rock mass joint surface after n shearing cycles can be expressed as follows:

20 $$\left\{\begin{array}{l}{\tau }_{n}=\gamma \left(t\right)\cdot {\sigma }_{n}tan(2{\alpha }_{n}+{\varphi }_{n})\\ {\alpha }_{n}={\alpha }_{0}{R}_{s}={\alpha }_{0}\left[A\left({e}^{-n/B}-1\right)+1\right]\\ {\varphi }_{n}=\frac{\left({\alpha }_{0}-{\alpha }_{n}\right)\left[\text{arctan}\left({\tau }_{r}/{\sigma }_{n}\right)-{2\alpha }_{r}\right]+\left({\alpha }_{n}-{\alpha }_{r}\right){\varphi }_{0}}{{\alpha }_{0}-{\alpha }_{r}}\\ \gamma \left(t\right)={\gamma }_{r}+(1-{\gamma }_{r}){e}^{-a\left|\dot{x}\left(t\right)\right|}\end{array}\right..$$

## Case study

### Case 1

#### Parameters

The cyclic shear test results of ten joint surfaces with different initial undulant angles (16°, 25°, 33°, 40°, and 45°), joint wall compressive strengths (10 MPa, 20 MPa, and 30 MPa), and initial basic friction angles (28.6°, 29.3°, and 30.5°) under different normal stresses (0.8, 1.6, 2.4, and 3.2) from Liu^35,36^ were used to verify the feasibility of the proposed method in this study. The shear rate is 0.5 mm s^−1^. And the calculation parameters for the cyclic shear strengths of the joints are listed in Table 2.

**Table 2:** Calculation parameters for cyclic shear strength of joints.

No	${\alpha }_{0}$/(°)	${\sigma }_{c}$/MPa	${\sigma }_{n}$/MPa	${\varphi }_{0}$/(°)	${\tau }_{r}$/MPa
J46	16	30	1.6	30.5	0.798
J16	16	10	3.2	28.6	1.228
J22	25	20	0.8	29.3	0.449
J37	25	20	3.2	29.3	1.165
J03	33	10	0.8	28.6	0.436
J23	33	20	0.8	29.3	0.449
J14	40	10	2.4	28.6	0.945
J39	40	20	3.2	29.3	1.196
J10	45	10	1.6	28.6	0.630
J40	45	20	3.2	29.3	1.196

#### Calculation of dilatancy angle and basic frictional angle

In Eq. (10), the calculation coefficients A and B of the joint surfaces with different initial undulation angles are different and should be fitted according to the shear test results of the joint surfaces with each initial undulation angle. However, because many types of joint surfaces with different initial undulation angles are involved in the calculation process, the solution is complicated. During variation trend analysis of the dilatation angle with the number of cyclic shears in “Dilatancy angle of the joint surface” section, the dilatation angle curve with the number of joint surface shear cycles with an initial undulation angle of 16° was in the middle position, which was roughly representative. In this study, the experimental values of joint samples with an initial undulation angle of 16° were used as a typical case to describe the solution process of the dilatation angle in detail. The calculation process of the empirical equation of the shear dilatancy angle of joint surfaces with other undulation angles was the same as in the typical case.

**Figure 6:**
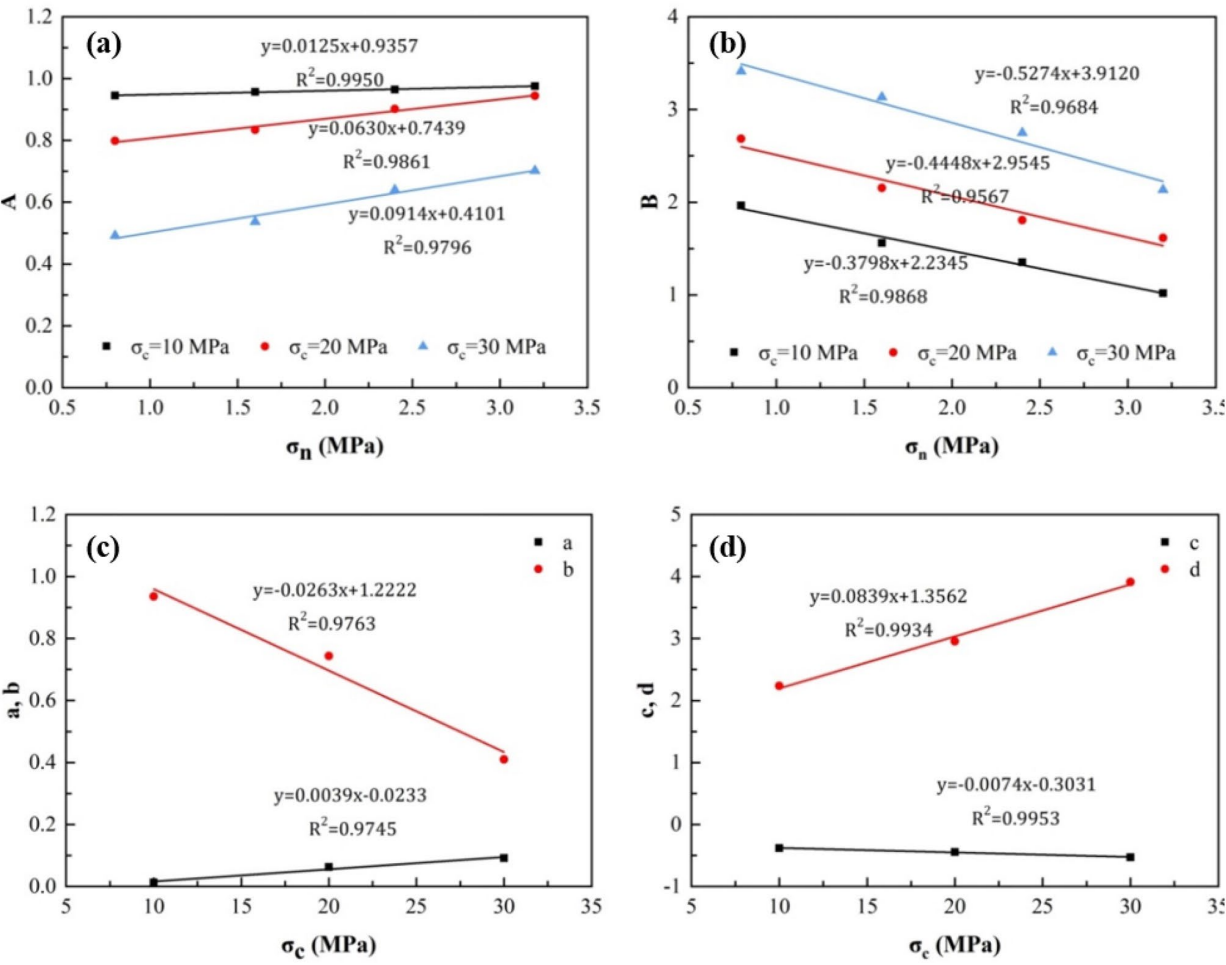
Relationship curves between parameters. (**a**) Relationship curves between parameter A and ${\sigma }_{n};$ (**b**) relationship curves between parameter B and ${\sigma }_{n}$; (**c**) relationship curves between parameter a, b and ${\sigma }_{c}$; and (**d**) relationship curves between parameter c,d and ${\sigma }_{c}$.

According to the experimental results of the dilatancy angle coefficient ($${R}_{s})$$ for the joint surface sample with an initial undulation angle of 16° (Table 3), fitting the data to Eqs. (12) and (13) allowed us to obtain the correlation coefficients *A* and *B* related to Eq. (11), and the relationship between parameters *A* and *B* and the normal stress of the joint surface with different intact rock strengths, as shown in Fig. 6a,b. The relationships between *a*, *b*, *c*, and *d* and the intact rock strength $${\sigma }_{c}$$ were regressed, and the results of this analysis are shown in Fig. 6c,d, and the expressions of *A* and *B* can be determined as follows:

**Table 3:** Test results of ${R}_{s}$ for joint surface sample with initial asperity angle of 16° ^36^.

${\sigma }_{c}=10\, \text{MPa}$					${\sigma }_{c}=20\, \text{MPa}$				${\sigma }_{c}=30\, \text{MPa}$			
Sample number	${\sigma }_{n}$											
	0.8 MPa	1.6 MPa	2.4 MPa	3.2 MPa	0.8 MPa	1.6 MPa	2.4 MPa	3.2 MPa	0.8 MPa	1.6 MPa	2.4 MPa	3.2 MPa
0	1.00	1.00	1.00	1.00	1.00	1.00	1.00	1.00	1.00	1.00	1.00	1.00
1	0.651	0.577	0.516	0.404	0.739	0.650	0.602	0.577	0.901	0.880	0.818	0.716
2	0.398	0.288	0.224	0.141	0.643	0.479	0.383	0.310	0.776	0.739	0.654	0.555
3	0.245	0.175	0.140	0.075	0.560	0.386	0.276	0.200	0.703	0.656	0.566	0.478
4	0.163	0.118	0.101	0.053	0.494	0.325	0.212	0.146	0.657	0.611	0.514	0.425
5	0.123	0.081	0.069	0.043	0.440	0.272	0.174	0.106	0.620	0.573	0.469	0.384
6	0.099	0.065	0.051	0.030	0.378	0.231	0.142	0.079	0.592	0.541	0.441	0.353
7	0.085	0.055	0.038	0.026	0.331	0.199	0.121	0.063	0.573	0.525	0.417	0.329
8	0.075	0.053	0.036	0.021	0.283	0.170	0.107	0.056	0.556	0.504	0.393	0.312
9	0.068	0.049	0.032	0.019	0.253	0.162	0.091	0.061	0.544	0.494	0.382	0.297
10	0.069	0.046	0.031	0.019	0.236	0.160	0.080	0.059	0.535	0.484	0.369	0.283

21 $$A=\left(0.0039{\sigma }_{n}-0.0263\right){\sigma }_{c}-0.0233{\sigma }_{n}+1.2222,$$

22 $$B=\left(0.0839-0.0074{\sigma }_{n}\right){\sigma }_{c}-0.3031{\sigma }_{n}+1.3562.$$

Therefore, according to Eq. (10), the calculation equation of the dilation angle in this case was calculated as follows:

23 $$\left\{\begin{array}{l}{\qquad\alpha }_{n}={\alpha }_{0}\left[A\left({e}^{-n/B}-1\right)+1\right]\\ A=\left(0.0039{\sigma }_{n}-0.0263\right){\sigma }_{c}-0.0233{\sigma }_{n}+1.2222.\\ B=\left(0.0839-0.0074{\sigma }_{n}\right){\sigma }_{c}-0.3031{\sigma }_{n}+1.3562\end{array}\right.$$

Based on the dilation angle obtained by solving according to Eq. (23), the basic friction angle of the structural surface after each cycle could be obtained using Eq. (17).

#### Calculation results

**Figure 7:**
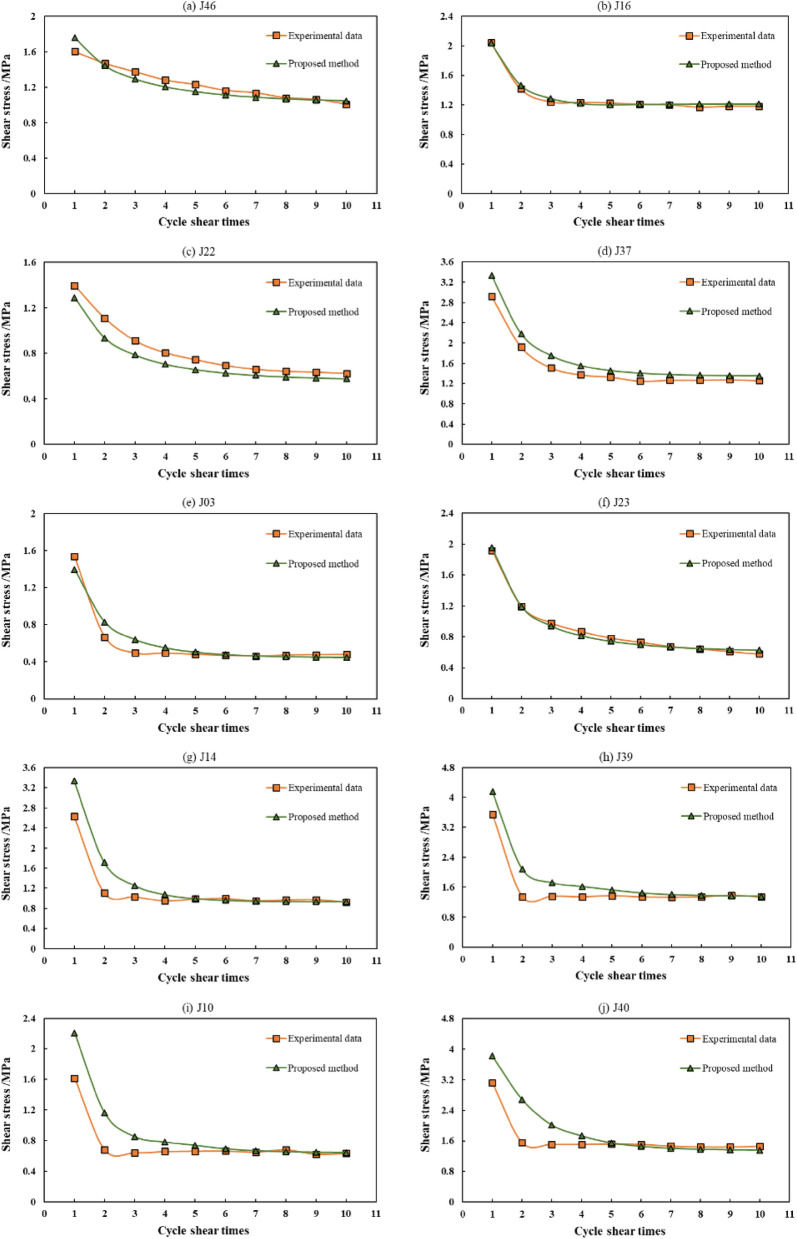
Comparison between the calculated results and the experimental values.

A comparison between the calculated and experimental values is presented in Fig. 7. For the samples with medium and low undulation angle joint surfaces, the calculated values of shear strength of joints with different strength grades gradually decreased with an increase in the number of cycles, and the decreasing trend gradually slowed down, which is in good agreement with the experimental results. For the joint specimens with high undulation angles (40° and 45°), although the change in calculated shear strength and the experimental value were basically the same, the calculated values of the shear strength of cycles 1 and 2 deviated substantially from the experimental value. Starting from cycle 3, the calculated values were consistent with the experimental values as the number of shear cycles increased. It is believed that the failure mode of joint specimens with a high undulation angle is different from that with a low undulation angle. For the high-undulation-angle-joint specimen, the protrusion failure mode was tensile rather than shear, and the tensile strength of the material was the controlling factor, which was inconsistent with the assumption that the matrix material reached the shear strength and failed in the calculation equation. Therefore, when using the calculation equation to estimate the cyclic shear strength of a joint, the sum of the convex angle of the joint surface and the basic friction angle of the surface should not be greater than 70°.

### Case 2

#### Experimental method

**Figure 8:**
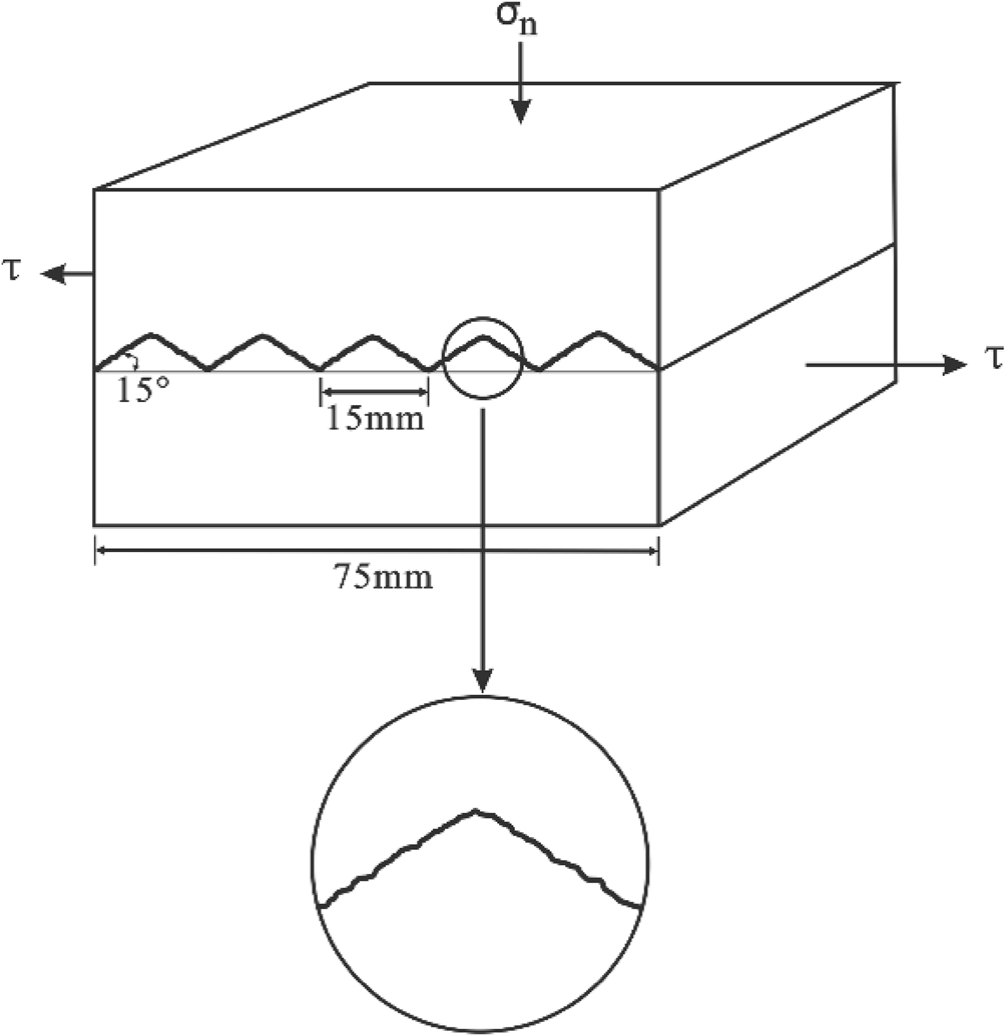
Sawtooth samples for direct shear test.

Jafari et al.^5^ used cement mortar and a silicone rubber moulding method to prepare a cubic sawtooth joint surface, and conducted a study on the shear strength of rock joints under cyclic loading. In a study by Jafari et al.^5^, the step length of the sawtooth protrusion was 15 mm, the degree of undulation was 15°, and many micro protrusions were set on the undulation surface (Fig. 8). The BCR-3D direct shear test device in the 3S Laboratory (Lab. 3S) at University Joseph Fourier in Grenoble, France, was used to perform the cyclic shear test of the sawtooth joint surface. The relative displacement applied in each shear cycle was 15 mm, and the upper and lower parts of the specimen moved 7.5 mm each. The tests were carried out at normal stress levels of 1.2 MPa and 6.5 MPa. The uniaxial compression strength of the samples was more than 55 MPa. The shear rate was in the range of 0.05–0.4 mm s^−1^.

#### Jafari proposed method

According to the experimental results analysis, Jafari et al.^5^ proposed a shear strength model of the structural surface specimen during a large cyclic shear displacement, which is:

24 $$\frac{\tau }{{\sigma }_{n}}=\frac{b{({NC}_{d})}^{p}{({i}_{n})}^{q}+c}{1+b{({NC}_{d})}^{p}{({d}_{n})}^{q}},$$

where $\tau$ is the shear strength, ${\sigma }_{n}$ is the normal stress, ${NC}_{d}$ is the number of displacement cycles, ${i}_{n}$ is the normalised dilation angle, and ${d}_{n}$ is the normalised degradation. The parameters b, c, p, and q are related to the mechanical properties of the tested sample and the geometrical characteristics of the structural surface. In this relation, parameters b, c, p, and q were obtained by model calibration as follows:

25 $$\left\{\begin{array}{l}b=-0.33\\ c =1.44\\ p=0.12\\ q=0.3\end{array}.\right.$$

#### Calculation results and comparative analysis

**Figure 9:**
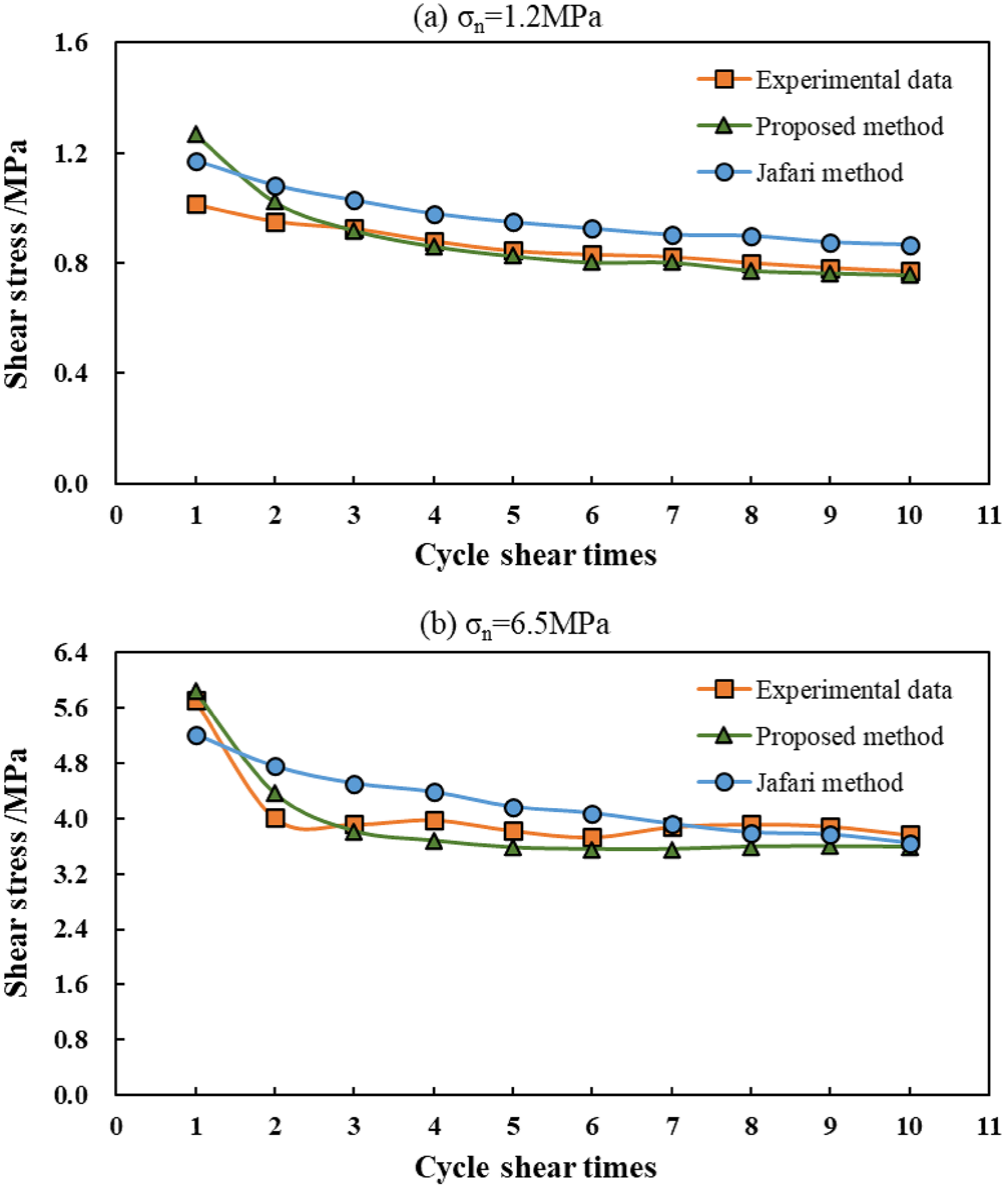
Comparison between the results of proposed method in this study, Jafari et al.^5^ proposed method, and the tests.

Because the data in the paper of Jafari et al.^5^ is not very comprehensive, and in the tests of Liu et al.^35^ and Jafari et al.^5^, the first-order undulation angle of the sample is very close, the Eq. (23) are used to calculate the dilatancy angle during the shear process. The average shear rate 0.225 mm s^−1^ and the intact rock strength 60 MPa are used to calculate the results in this case study. The results obtained by the method proposed in this study, Jafari et al.^5^ proposed method, and the tests are shown in Fig. 9. When the normal stress was 1.2 MPa, the shear stress obtained by Jafari et al.^5^ proposed method was slightly higher than the experimental results, and the shear stresses obtained by the method proposed in this study were in very good agreement with the experimental results, except for the value of cycle 1. When the normal stress was 6.5 MPa, the results obtained by the method proposed in this study, Jafari et al.^5^ proposed method, and the tests were very close; however, the variation in shear strength with the increase in cyclic shearing times of the method proposed in this study was closer to the experimental results, and the error of the method of comparison proposed by Jafari et al.^5^ was smaller. This shows that the calculation method proposed in this study is feasible and can also be used for structural surfaces containing asperities.

## Conclusion

This study discusses a study of the shear strength of a rock mass joint surface under cyclic loading, starting with the effect of cyclic shear deterioration on the joint surface in the rock mass and the reduction in the dynamic friction coefficient between sliding rock blocks caused by relative velocity, and proposes an equation for calculating the shear strength of a rock mass joint surface under cyclic shear loading. The following conclusions can be drawn from this investigation:

1. The dilatancy angle degradation, undulation wear, and shear rate effect on the friction angle are the three main factors affecting the shear strength of rock joints under cyclic loading.
2. The model proposed in this study is in good agreement with the experimental results and can also be used to calculate the shear strength of structural surface samples containing numerous asperities.
3. When the calculation formula is used to estimate the cyclic shear strength of the structural surface where the sum of the initial undulation angle and the basic friction angle is greater than 70°, there may be some errors in the calculation results.

## Data Availability

The data that support the findings of this study are available on request from the corresponding author.

## List of symbols

$L$: Total length of the joint surface sample
$l_{1}$: Fixed step length of the sawtooth protrusion
$l_{2}$: Fixed step length of the edge sawtooth protrusion
$\theta$: First-order undulation angle
$H$: Height of the sawtooth protrusions
$D_{n}$: Joint surface shear strength ratio
$\tau_{n}$: Peak shear strength of the *n-*th shear cycle
$\tau_{1}$: Peak shear strength of the first shear cycle
τ: Peak shear stress
$$\sigma_{n}$$: Normal stress
*i*: Average deviation angle of particle displacements from the applied shear stress direction
$$c$$: Cohesion
$\varphi$: Total friction angle
$JRC$: Joint roughness coefficient
$JCS$: Joint wall compressive strength
$\varphi_{b}$: Basic friction angle
$\alpha_{p}$: Peak dilatancy angle
$S_{a}$: Asperity failure component
$\alpha_{n}$: Dilatancy angle of the n-th cyclic shear
$\alpha_{0}$: Initial undulation angle
*n*: Number of shear cycles
$R_{s}$: Wear coefficient of the dilatancy angle
$\alpha_{r}$: Residual dilatancy angle
$\varphi_{0}$: Initial basic friction angle
$\varphi_{n}$: Basic friction angle of the joint surface of the n-th cyclic shear
$\varphi_{r}$: Residual basic frictional angle
${\uptau }_{{\text{r}}}$: Residual shear stress
$f_{c} \left( {\dot{x}} \right)$: A function that decreases with an increase in $\left| {\dot{x}} \right|$
$f_{s}$: Starting frictional coefficient that is related to cumulative displacement
$\dot{x}$: Relative velocity of the upper block relative to the base
${\upgamma }\left( t \right)$: Relative velocity factor
$\gamma_{r}$: Convergence value of the relative velocity influence factor
$NC_{d}$: Number of displacement cycles
$i_{n}$: Normalized dilation angle
$$d_{n}$$: Normalized degradation
$\sigma_{c}$: Intact rock strength
